# Whole Genome Sequence Analysis of Phage-Resistant *Listeria monocytogenes* Serotype 1/2a Strains from Turkey Processing Plants

**DOI:** 10.3390/pathogens10020199

**Published:** 2021-02-13

**Authors:** Phillip Brown, Yi Chen, Cameron Parsons, Eric Brown, Martin J. Loessner, Yang Shen, Sophia Kathariou

**Affiliations:** 1 Department of Plant and Microbial Biology, North Carolina State University, Raleigh, NC 27695, USA; pebrown4@ncsu.edu; 2 Division of Microbiology, Center for Food Safety and Applied Nutrition, Food and Drug Administration, College Park, MD 20740, USA; Yi.Chen@fda.hhs.gov (Y.C.); Eric.Brown@fda.hhs.gov (E.B.); 3 Department of Food, Bioprocessing and Nutrition Sciences, North Carolina State University, Raleigh, NC 27695, USA; ctparson@ncsu.edu; 4 Laboratory of Food Microbiology, Institute of Food, Nutrition and Health, ETH Zurich, Schmelzbergstrasse 7, CH-8092 Zurich, Switzerland; martin.loessner@ethz.ch (M.J.L.); yang.shen@hest.ethz.ch (Y.S.)

**Keywords:** *Listeria*, bacteriophage, phage resistance, whole genome sequencing, food processing plant, InlA, serotype 1/2a, wall teichoic acid

## Abstract

*Listeria* monocytogenes is a Gram-positive bacterial pathogen and the causative agent of listeriosis, a severe foodborne infection. *L. monocytogenes* is notorious for its ability to persist in food processing environments (FPEs) via a variety of adaptive traits. Even though traits such as cold tolerance, biofilm formation and sanitizer resistance have been extensively investigated for their roles in persistence of *L. monocytogenes* in FPEs, much less is known about resistance to bacteriophages. Previous studies explored phage resistance mechanisms in laboratory-created mutants but it is imperative to investigate phage resistance that is naturally exhibited in FPE-derived strains. Here, we integrated the analysis of whole genome sequence data from a panel of serotype 1/2a strains of sequence types 321 and 391 from turkey processing plants, with the determination of cell surface substituents required for phage adsorption and phage infection assays with the four wide-host-range phages A511, P100, 20422-1 and 805405-1. Using a specific set of recombinant phage protein probes, we discovered that phage-resistant strains lacked one or both of the serogroup 1/2-specific wall teichoic acid carbohydrate decorations, *N*-acetylglucosamine and rhamnose. Furthermore, these phage-resistant strains harbored substitutions in *lmo1080*, *lmo1081*, and *lmo2550*, which mediate carbohydrate decoration of the wall teichoic acids.

## 1. Introduction

*Listeria monocytogenes* is a Gram-positive facultative intracellular pathogen found ubiquitously in nature and is notorious for its capacity to persist in food processing environments (FPEs). Contamination of FPEs is critical for contamination of ready-to-eat foods by *L. monocytogenes*, with the potential to lead to outbreaks of the severe and potentially life-threatening foodborne disease listeriosis [1,2,3]. *L. monocytogenes* has the ability to persist in FPEs via multiple adaptations, including its ability to grow at low temperatures, to form biofilms and tolerate sanitizers [4,5,6,7,8]. Even though such adaptive traits have been extensively investigated, the potential roles of bacteriophage (phage) resistance in the persistence of this pathogen in FPEs remains poorly understood.

*Listeria*-specific phage have been approved as a biocontrol tool against *Listeria* in FPEs and foods, e.g., phage P100 in Listex P100 [9]. Repeated interactions between *L. monocytogenes* and phage exert selective pressures that may select for, and eventually result in, phage resistance. Phage resistance in *L. monocytogenes* can be mediated by failure of the phage to adsorb to its specific receptors via receptor loss or modification, and various post-infection intracellular resistance mechanisms [10,11,12]. In *L. monocytogenes* and other bacteria, the latter can include prophages, bacteriophage exclusion (BREX) systems, defense island system associated with restriction-modification (DISARM) systems and clustered, regularly interspaced, short palindromic repeats (CRISPR) systems [13,14,15,16,17,18,19].

Investigations of phage resistance established under laboratory conditions have shown that certain genes of *L. monocytogenes* are critical both for normal wall teichoic acid (WTA) decoration and for phage susceptibility [10,11,20,21,22]. In strains of serogroup 1/2, the WTA decorations required for susceptibility to broad-host-range phages such as A511 and P100 are *N*-acetylglucosamine and rhamnose [23,24,25,26]. Many of the genes mediating phage susceptibility in serotype 1/2a are found in two chromosomal operons, *lmo1079-lmo1084* and *lmo2549-lmo2550* [10,25,27]. A previous study showed that spontaneous phage-resistant mutants of *L. monocytogenes* 10403S harbored mutations concentrated in two loci containing seven genes in total (*lmo1079-lmo1084* and *lmo2549-lmo2550*). Furthermore, serotype 1/2a isolates from seafood industries frequently harbored non-synonymous mutations in *lmo2549* or *lmo2550* [14]. However, reports that integrate phage resistance, WTA decoration analysis and underlying genetic alterations in *L. monocytogenes* under field conditions, such as prevailing in FPEs, are largely lacking.

In a previous study, we characterized *L. monocytogenes* from different turkey processing plants in the United States for their resistance to a panel of phages [12]. The majority of the phage-resistant strains were of serotype 1/2a, followed by 1/2b and 1/2c, i.e., serotypes which are commonly encountered in food and food processing ecosystems [12,28,29,30,31]. In the current study, our objective was to integrate whole genome sequence analysis, phage adsorption assays and phenotypic characterization of WTA decorations in order to elucidate mechanisms mediating phage resistance in FPE-derived serotype 1/2a strains. To address this objective, we utilized whole genome sequence data to assess genomic differences between phage-resistant and phage-susceptible strains, as well as to identify sequence alterations in a targeted panel of genes previously implicated in WTA biosynthesis. These data were further correlated with the WTA decoration patterns revealed by a novel set of glycotyping protein probes [32] and with phage adsorption and infection assays.

## 2. Results and Discussion

Earlier investigations characterized isolates of L. monocytogenes from turkey processing plants in the US for resistance to phage as well as to benzalkonium chloride and the heavy metals cadmium and arsenic [12,33]. A subset of 10 strains of serotype 1/2a that were previously screened for resistance to three wide-host-range phages, i.e., 20422-1, 805405-1 and A511 and were also resistant to benzalkonium chloride and cadmium were chosen for whole genome sequencing (Table 1). *In-silico* multilocus sequence typing (MLST) revealed two sequence types (STs), ST321 and ST391 (Table 1). Strains of the same ST were repeatedly isolated from the same processing plant over a 30-month period (Table 1), reflecting potential persistence in the FPE. In a previous study of South African food products, 9.7% of *L. monocytogenes* isolates belonged to ST321, while in another study 33 of 42 isolates of *L. monocytogenes* from a cold-smoked salmon processing facility belonged to ST or CC321 [34]. Analysis of the ST321 strains used in the current study (Table 1) using the NCBI pathogen detection pipeline [35] identified numerous similar strains from food and environmental isolates (data not shown). In contrast to ST321, little is known about ST391 in the food processing environment. Analysis of the ST391 strains analyzed in this study (Table 1) using the NCBI pathogen detection pipeline identified only a small number of closely-related strains, from ready-to-eat foods [35]. 

The previously-reported phage resistance profiles [12] were confirmed for all strains, which were additionally tested for their susceptibility to the broad-host-range phage P100. While phages 20422-1 and 805405-1 were isolated from turkey processing plants in the United States in the same study as the *L. monocytogenes* strains investigated here [12], phages A511 and P100 were isolated in Germany in the 1990s [36,37,38,39]. All tested strains were either resistant (R) or susceptible (S) to all four phages, with only one strain of each ST being susceptible (strains L1624a and #24 in ST321 and ST391, respectively) (Table 1). Hereafter, the terms phage resistance and susceptibility pertain to the observed resistance or susceptibility, respectively, towards the four wide-host-range phages that were employed, i.e., A511, P100, 20422-1 and 805405-1.

The WGS data of all strains were also analyzed for the presence of premature stop codons (PMSCs) in *inlA*, previously reported to be common among serotype 1/2a strains from FPEs and associated with hypovirulence [27,29,40,41]. All six strains of ST321 harbored a shared PMSC (T730) in *inlA*, regardless of whether they were phage-resistant or susceptible (Table 1). PMSCs in *inlA* were also found in ST321 strains from the South African food study as well as a cold-smoked salmon processing facility [34,42], suggesting that this is a clonal trait potentially reflecting adaptation of these strains to the processing plant environment. None of the ST391 strains harbored PMSCs in *inlA* (Table 1).

### 2.1. Absence of WTA Substituents in Phage-Resistant FPE-Derived Strains of ST321 and ST391 Is Accompanied by Phage Resistance

**Figure 1:**
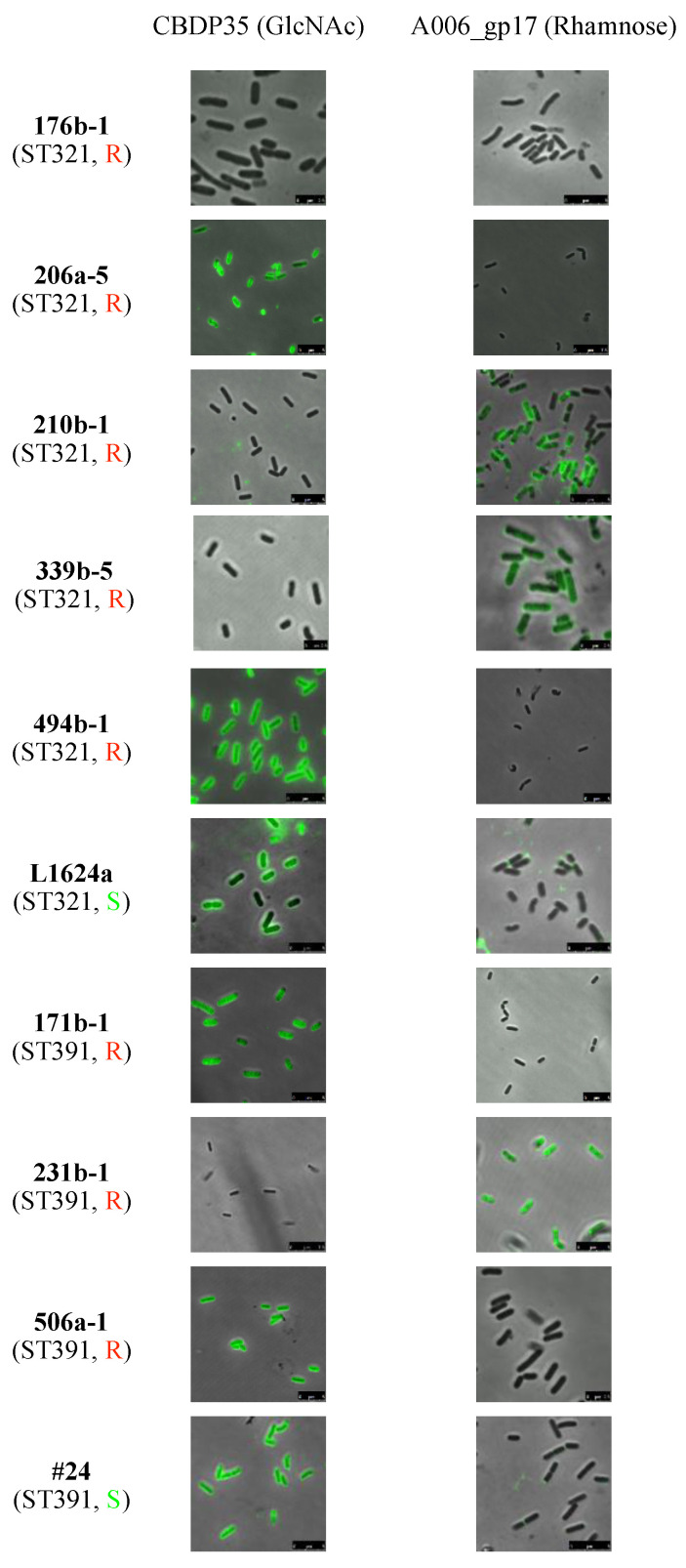
Glycotyping of serotype 1/2a *L. monocytogenes* strains of ST321 and ST391 strains investigated in this study. *N*-acetylglucosamine (GlcNAc) and rhamnose (Rhamn) were detected using GFP-labeled CBDP35 and A006_gp17 [32]. The green signal indicates the presence of the respective WTA substituent. Sequence type (ST) designations are in parentheses underneath the strain designation. R (red font) and S (green font) indicates phage resistance and susceptibility, respectively, to the four wide-host-range phages employed in the study, i.e., A511, P100, 20422-1 and 805405-1. The glycotyping assay was done as described in Materials and Methods.

Previous studies have shown that *N*-acetylglucosamine (GlcNAc) and rhamnose substituents on WTA are critical for adsorption of phages A118, A511 and P35 to *L. monocytogenes* of serotype 1/2a, with loss of either of these conferring phage resistance [39,43,44,45]. Employment of a glycotyping assay specific to WTA-associated GlcNAc and rhamnose [32] revealed that the phage-susceptible strains L1624a and #24 (ST321 and, ST391, respectively) were positive for both GlcNAc and rhamnose, with the latter exhibiting relatively weak binding concentrated at the polar ends of the cell (Table 1 and Figure 1). In contrast, all phage-resistant strains of ST321 and ST391 lacked at least one of the WTA substituents. Specifically, the ST321 strains 339b-5 and 210b-1 and the ST391 strain 231b-1 were missing GlcNAc, while the ST321 strains 206a-5 and 494b-1 and the ST391 strains 171b-1 and 506a-1 were lacking rhamnose. Interestingly, one strain, 176b-1 (ST321) was missing both of these WTA substituents (Table 1 and Figure 1). The absence of both GlcNAc and rhamnose in the WTA of this strain was surprising. There is no obvious selective pressure for the loss of both WTA substituents, as the absence of just one is sufficient for resistance to broad-host-range phage [43,44]. Further studies are needed to elucidate the potential selective pressures that may render the loss of both WTA substituents advantageous to this strain.

**Figure 2:**
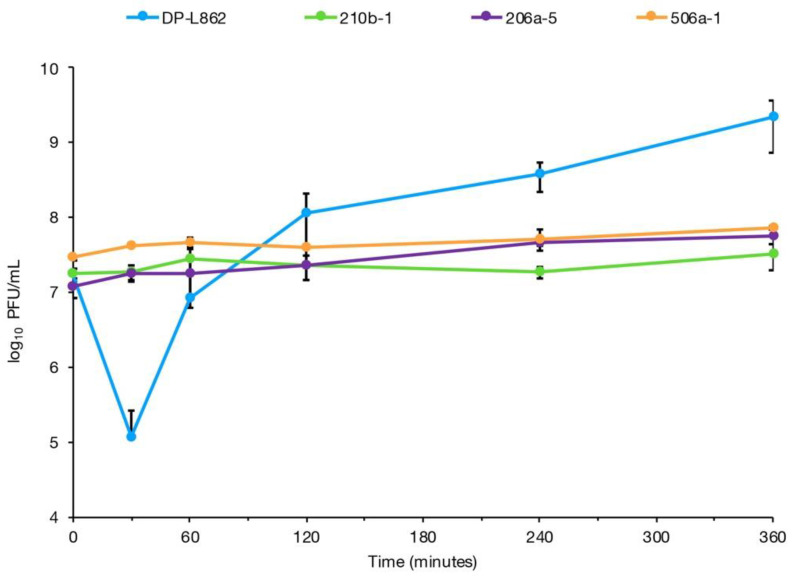
Failure of phage to adhere to representative phage-resistant serotype 1/2a *L. monocytogenes* strains of ST321 and ST391. The phage-resistant strains were exposed to phage P100 for 360 min and PFU/mL in the supernatant was enumerated at specific time points as described in Materials and Methods. The phage-susceptible serotype 1/2a strain *L. monocytogenes* DP-L862 (blue) was used as positive control and the phage-resistant strains are 210b-1 (ST321, green), 206-5 (ST321, purple) and 506a-5 (ST391, orange). Error bars represent standard deviation.

Previous work reported that phage 20422-1 failed to adsorb on strain 176b-1 (ST321) [12]. This was supported by the glycotyping data discussed above, which showed absence of both GlcNAc and rhamnose in this strain (Figure 1, Table 1). Testing phage P100 adsorption on additional strains of each ST representing the remaining glycotyping profiles (Table 1) confirmed that the phage failed to adsorb on phage-resistant strains that lacked either GlcNAc or rhamnose (Figure 2).

### 2.2. FPE-Derived Strains Are Closely Related Despite Differences in Phage Resistance and Glycotyping Profiles

**Figure 3:**
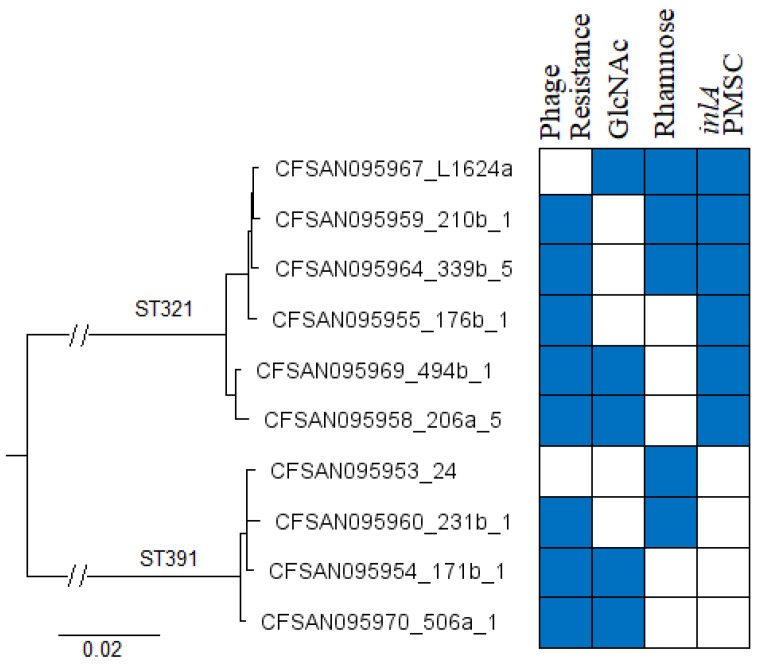
Phylogenetic tree of FPE-derived phage-resistant and phage susceptible *L. monocytogenes* strains investigated in this study. Phage resistance (blue) and susceptibility (white), presence (blue) or absence (white) of *N*-acetylglucosamine (GlcNAc) and rhamnose in the WTA, and presence (blue) or absence (white) of an *inlA* premature stop codon (PMSC) are indicated in the heat map to the right of the tree. The strain designations are included after the CFSAN designation (with hyphens replaced by _), e.g. the top first and second strains in the tree correspond to strains L1624a and 210b-1, respectively. The tree was based on 1748 *L. monocytogenes* core genes from the Institute Pasteur multilocus sequence typing (MLST) database and was constructed as described in Materials and Methods.

Analysis of the WGS data revealed that the FPE strains of the same ST were closely related (Figure 3). Analysis of the core genome (1748 genes) revealed only four to 18 cgMLST differences among the ST321 strains, and even fewer (four to eight cgMLST differences) among those of ST391. Interestingly, these strains did not segregate phylogenetically by their phage resistance phenotypes or glycotyping profiles (Figure 3). For instance, the ST321 strains L1624a and 210b-1 that were phage susceptible and resistant, respectively, only had four cgMLST allele differences (*lmo0135*, *lmo0947*, *lmo1711* and *lmo2518*).

WTA substituents are serotype-specific [23] and the genes involved in WTA glycosylation, e.g., *lmo1079-lmo1084* and *lmo2549-lmo2550*, are not part of the *L. monocytogenes* core genome. Therefore, whole genome MLST (wgMLST) was also employed to determine allele differences among the strains. A relatively small number of differences were also found using wgMLST analysis, with 14–106 wgMLST differences among ST321 strains and only 16–46 wgMLST differences among those of ST391. Based on wgMLST there are 66 variable genes among the ST321 and 35 among the ST391 strains, excluding missing and incomplete alleles (Supplementary Table S1, Supplementary Table S2). Comparison of the alleles found to be variable by wgMLST revealed only three genes that were variable in both STs: *lmo1080*, *lmo1081* and *lmo2550*. As indicated earlier, these three genes have all been implicated in WTA glycosylation, either with rhamnose (*lmo1080* and *lmo1081*) or *N*-acetylglucosamine (*lmo2550*) [10,14,25,43].

### 2.3. SNPs in Wall Teichoic Acid Genes Contribute to Phage Resistance in FPE-Derived Strains

**Figure 4:**
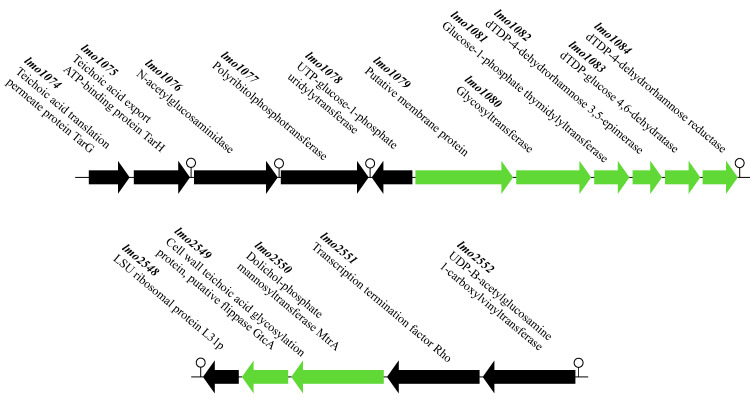
Genomic organization of *lmo1079-lmo1084* (top) and *lmo2549*-*lmo2550* (bottom) in the serotype 1/2a reference strain *L. monocytogenes* EGD-e [46]. Arrows indicate the direction of transcription. Green indicates ORFs previously described to be involved in glycosylation of WTA in *L. monocytogenes* serotype 1/2a with *N*-acetylglucosamine or rhamnose [10,14]. Lollipop symbols indicate putative terminators.

In a previous study, phage resistance of *L. monocytogenes* 10403S selected under laboratory conditions was found to be accompanied by SNPs in a panel of genes mediating glycosylation of the WTA with rhamnose and *N*-acetylglucosamine [10]. These genes are organized in two operons, *lmo1079-1084* and *lmo2549-lmo2550*, in the chromosome of *L. monocytogenes* serotype 1/2a (Figure 4). Interestingly, the only three genes found to be variable in both STs via the wgMLST analysis discussed above, i.e., *lmo1080*, *lmo1081* and *lmo2550*, belonged to these two operons.

To determine whether mutations in the genes of the *lmo1079-1084* and *lmo2549-lmo2550* operons were associated with phage resistance in the FPE-derived-resistant strains in the current study, we compared the sequence of each gene between phage resistant and phage-susceptible strains of the same ST. Two phage-resistant strains, 494b-1 (ST321) and 506a-1 (ST391), were found to have non-synonymous SNPs in *lmo1080* and to lack rhamnose in the WTA (Table 1, Figure 1). Disruption of *lmo1080*, as well as the dTDP-L-rhamnose biosynthesis genes (*lmo1081*-*lmo1084*), have been shown to result in loss of rhamnose in the WTA, as well as phage resistance [10,25]. In addition, phage P100 failed to adsorb to strain 506a-1 (Figure 2). Four strains, i.e., the ST321 strains 176b-1, 339b-5 and 210b-1 and the ST391 strain 231b-1, were found to have non-synonymous SNPs in *lmo2550* and also lacked GlcNAc in the WTA (Table 1, Figure 1). Previous and current adsorption assays with two of these strains, 176b-1 and 210b-1, indicated failure of phage to adsorb to 176b-1 [12] and 210b-1 (Figure 2). Mutations in *lmo2550* in serogroup 1/2 strains have been known to be accompanied with lack of GlcNAc in the WTA, both in laboratory mutants and seafood industry strains, even though resistance of the latter to phage was not reported [14,43].

Two strains, 206a-5 (ST321) and 171b-1 (ST391), were found to harbor PMSCs in *lmo1080* and were missing rhamnose from the WTA. While no other SNPs were found in 171b-1 there was a second SNP in 206a-5 (*lmo1084*). The latter SNP is not expected to cause the loss of rhamnose as it was also found in four other strains, two of which had rhamnose in the WTA (Table 1, Figure 1). While not causing the absence of rhamnose or GlcNAc from the WTA, this non-synonymous substitution at nt 599 of *lmo1084* was found in all five phage-resistant ST321 strains.

The SNP and glycotyping data, together with findings from the previous literature [10,14], allow us to postulate that PMSCs and non-synonymous SNPs in *lmo1080* and *lmo1081* may cause absence of rhamnose from the WTA, while non-synonymous SNPs in *lmo2550* may cause absence of GlcNAc from the WTA in *L. monocytogenes* colonizing food processing plants, leading to resistance to wide-host-range phages. Phage adsorption data for strains 206a-5, 506a-1, 210b-1 and 176b-1 suggest linkage between mutations in *lmo1080*, *lmo1081*, and *lmo2550* and failure of P100 to adsorb to the cell (Figure 2). Interestingly, the SNPs in these genes were at different locations than those reported previously [10], suggesting that mutations in multiple locations of these genes can alter WTA decoration and lead to phage resistance.

### 2.4. Further Studies

Exposure to phage in the FPEs and other environments may select for the loss of teichoic acid decorations in *L. monocytogenes*. The resulting resistance to phage may contribute to the apparent FPE persistence of the ST321 and ST391 strains investigated here, which were closely related and recovered from the same FPE over more than two years. However, in addition to serving as phage receptors, WTA glycosylation is increasingly recognized for its importance in other functions including surface adhesion, biofilm formation, anchoring of virulence determinants to the cell surface and resistance to antimicrobial peptides [14,19,47,48,49]. It will be of interest to investigate such potential trade-offs with the strains investigated here and other FPE-derived phage-resistant strains of serotype 1/2a. WTA glycosylation profiles differ noticeably among different serotypes of *L. monocytogenes* [23,24], and the fitness impacts of the loss of WTA decorations may exhibit serotype-specific traits.

## 3. Materials and Methods

### 3.1. Bacterial Strains and Growth Conditions

The *L. monocytogenes* strains investigated in this study are listed in Table 1. Unless otherwise noted, *L. monocytogenes* strains were grown in brain heart infusion broth (BHI; Becton, Dickinson & Co., Sparks, MD, USA) at 37 °C or on Luria–Bertani (LB) supplemented with 1.2% agar (LBA; Becton, Dickinson & Co.) and 10 mM calcium chloride (CaCl_2_) at 25 °C.

### 3.2. Listeria Phage Collection and Propagation, Phage Susceptibility and Adsorption Assays

*Listeria* phages used in this study are listed in Table 2. Phage propagation was as described [12] using *L. monocytogenes* DP-L862 as the propagating strain, resulting in phage titers of approximately 1.0 × 10^9^ plaque forming units (PFU)/mL. Strains were screened for phage susceptibility in 96-well plates, as described [12], with minor modifications. Specifically, each strain was tested using six dilutions of phage, ranging from undiluted (~ 1.0 × 10^9^ PFU/mL) to 10^−5^ (~1.0 × 10^4^ PFU/mL). The 96-well plates were then incubated at 37 °C for 30 min, well contents transferred on to LBA-10mM CaCl_2_ using a stainless-steel replicator and incubated overnight at 25 °C.

Phage adsorption assays were done as described [12] with minor modifications. Specifically, *L. monocytogenes* DP-L862 was used to enumerate filtrate dilutions for each time point after phage infection (0, 0.5, 1, 2, 4 and 6 h). *L. monocytogenes* DP-L862 was used as positive control for phage adsorption and replication. Strains were tested in at least two independent trials.

### 3.3. Whole Genome Sequencing and Analysis

Genomic DNA was extracted using a DNeasy blood and tissue kit (Qiagen, Valencia, CA) from strains grown overnight at 37 °C in BHI broth (Becton, Dickinson & Co.). Libraries were prepared using 1 ng of genomic DNA with a Nextera XT DNA library preparation kit (Illumina, San Diego, CA, USA), and the genomes were sequenced using either a NextSeq 500 sequencer with the NextSeq 500/550 high-output kit v2.5 (300 cycles, 2 × 150 bp) (Illumina) or a MiSeq desktop sequencer with the Miseq kit v2 (500 cycles, 2 × 250 bp) (Illumina) according to the manufacturer’s instructions. The raw sequencing reads were then quality-trimmed and assembled *de novo* using Spades v.3.14.1 [50]; the assemblies were then quality-assessed using QUAST v.4.6.4 [51]. Default parameters were used for all software.

Whole genome analysis including an in-house BLAST of target genes was conducted using the Pathosystems Resource Integration Center (PatricBRC) and Artemis [52,53]. Briefly, chromosomal nucleotide locations of previously-described single-nucleotide polymorphisms (SNPs) were taken from Denes et al. [10] and localized in the *L. monocytogenes* 10403S genome using the nucleotide search function in Artemis. This was converted into whole-genome nucleotide locations for each of the genomes as well as ORF nucleotide locations for the target genes, yielding both whole-genome nucleotide location and nucleotide location within the relevant ORF (Table 1). We extracted the wild-type alleles of the genes of interest (*lmo1079-lmo1084*, *lmo2549-lmo2550*) from the phage-susceptible strains of each ST and used BLAST to identify their counterparts in phage-resistant strains of the same ST. Whole genome (wgMLST) and core genome (cgMLST) multilocus allele differences were identified using BIGSdb PasteurMLST Genome Comparator [54]. A phylogenetic tree of the strains was constructed by importing the 1748 core genes from the PasteurMLST into Ridom SeqSphere+, as previously described [55].

### 3.4. Glycotyping Protein Toolkit Analysis

Glycotyping with a pair of GFP-tagged phage proteins including A006_gp17 (to identify rhamnose) and CBDP35 (to identify *N*-acetylglucosamine) was performed as previously described [32]. Briefly, *Listeria* cells from log phase cultures (OD600nm = ~0.5) were harvested by centrifugation (10,000 g, 1 min), and resuspended in 1/5 volume of PBS (pH 7.4). The cell suspension (100 µL) was incubated with 5 µL of 1 mg/mL of the GFP-fused proteins and incubated for 5 min at room temperature. The cells were centrifuged, washed twice in PBS and finally resuspended in PBS. The samples were transferred onto a glass slide with a cover slip and examined by a confocal laser scanning microscope (Leica Microsystems GmbH, Germany) equipped with a HCX PL FLUOTAR 100 × 1.30 oil objective. Image analysis was performed in Leica Suite Software (Bitplane AG, Zurich, Switzerland). For ease of visualization, contrast in red and green channels was enhanced. Each strain was tested in at least two independent trials.

### 3.5. Genome Sequence Accession Numbers

The whole genome sequence for strains used in this study can be found under the following accession numbers under the BioProject PRNJA215355: 176b-1 (SRR13521630), 206a-5 (SRR13521680), 210b-1 (SRR13521681), 339b-5 (SRR13521793), 494b-1 (SRR13521790), L1624a (SRR13521632), 171b-1 (SRR13521825), 231b-1 (SRR13521718), 506a-1 (SRR13521795) and #24 (SRR13521631).

## Figures and Tables

**Table 1:** Food processing environment (FPE)-derived strains of *L. monocytogenes* used in this study.

Strain ^1^	CFSAN #	Serotype	ST	Source ^2^	Date ^3^	InlA ^4^	Phage ^5^	GlcNac ^6^	Rham ^6^	Gene(s)	SNP ^7^	Mutation ^8^
176b-1 *	CFSAN095955	1/2a	321	A	4/04	T730	R	-	-	*lmo2550* *lmo1084*	Nt895 (C to T), Nt 2,579,952 Nt599 (A to T), Nt 1,100,483	NS NS
206a-5 *	CFSAN095958	1/2a	321	A	6/04	T730	R	-	+	*lmo2550* *lmo1084*	Nt895 (C to T), Nt 2,579,952 Nt599 (C to T), Nt 1,100,483	NS NS
210b-1 *	CFSAN095959	1/2a	321	A	6/04	T730	R	-	+	*lmo2550* *lmo1084*	Nt895 (C to T), Nt 2,579,952 Nt599 (C to T), Nt 1,100,483	NS NS
339b-5	CFSAN095964	1/2a	321	A	12/04	T730	R	+	-	*lmo1080* *lmo1084*	Nt1165 (G to T), Nt 1,096,726 Nt599 (C to T), Nt 1,100,483	PMSC NS
494b-1	CFSAN095969	1/2a	321	A	3/06	T730	R	+	-	*lmo1081* *lmo1084*	Nt674 (G to A), Nt 1,098,123 Nt599 (C to T), Nt 1,100,483	NS NS
L1624a	CFSAN095967	1/2a	321	B	7/05	T730	S	+	+			
171b-1	CFSAN095954	1/2a	391	A	4/04	FL	R	+	-	*lmo1080*	Nt267 (G to A), Nt 1,095,828	PMSC
231b-1	CFSAN095960	1/2a	391	A	8/04	FL	R	-	+	*lmo2550*	Nt479 (G to A), Nt 2,580,368	NS
506a-1 *	CFSAN095970	1/2a	391	A	3/06	FL	R	+	-	*lmo1081*	Nt116 (C to T), Nt 1,097,565	NS
#24	CFSAN095953	1/2a	391	A	9/03	FL	S	+	+			

^1^ Strains tested for phage adsorption are marked with *. Strain 176b-1 was previously tested for phage adsorption using phage 20422-1 [12], while strains 206a-5, 210b-1 and 506a-1 were tested in this study with phage P100. ^2^ Strains were from different sites in two different turkey processing plants (A and B) in the United States, as previously described [12]. ^3^ Date is in month/year, as previously described [12]. ^4^ InlA length is indicated as either full length (FL; 800 AA) or by the position of the premature stop codon in the deduced polypeptide. ^5^ R and S indicate phage resistance and susceptibility, respectively, to the four wide-host-range phages A511, P100, 20422-1 and 805405-1. ^6^ *N*-acetylglucosamine and rhamnose are indicated by GlcNac and Rham, respectively. Their presence or absence is indicated by + and -, respectively. ^7^ Genes harboring mutations. SNPs in the corresponding gene are indicated by the SNP location in the ORF followed by location in the entire chromosome. ^8^ NS and PMSC indicate non-synonymous mutation and premature stop codon, respectively.

**Table 2:** Wide-host-range bacteriophages used in this study.

Phage	Characteristics ^1^	Source [Reference]	Date
20422-1	ND	Processing Plant, North Carolina, USA [12]	2004
805405-1	ND	Processing Plant, Virginia, USA [12]	2005
A511	Virulent, *Myoviridae*	Sewage, Germany [40]	1990
P100	Virulent, *Myoviridae*	Sewage, dairy plant, southern Germany [40]	1997

^1^ ND, not determined, as these phages have not been sequenced or fully characterized.

## Data Availability

Not applicable.

## Supplementary Materials

The following are available online at https://www.mdpi.com/2076-0817/10/2/199/s1, Table S1: ST321 wgMLST variable alleles, Table S2: ST391 wgMLST variable alleles.
